# Efficacy of chimeric antigen receptor T-cell therapy in testicular relapse of pediatric acute lymphoblastic leukemia: a multicenter retrospective study

**DOI:** 10.3389/fimmu.2026.1766494

**Published:** 2026-02-11

**Authors:** Ning Wang, Yanjing Tang, Yong Zhuang, Jiaoyang Cai, Shaoyan Hu, Xue Yang, Yongjun Fang, Xiaoyan Wu, Ningling Wang, Lingzhen Wang, Xiaowen Zhai, Minghua Yang, Xin Tian, Yaqin Wang, Hong Li, Leifeng Zhao, Chi Kong Li, Xiaojuan Chen, Xiaofan Zhu

**Affiliations:** 1 Department of Pediatric Hematology and Oncology, State Key Laboratory of Experimental Hematology, National Clinical Research Center for Blood Diseases, Haihe Laboratory of Cell Ecosystem, Institute of Hematology & Blood Diseases Hospital, Chinese Academy of Medical Sciences & Peking Union Medical College, Tianjin, China; 2 Tianjin Institutes of Health Science, Tianjin, China; 3 Department of Hematology/Oncology, Shanghai Children’s Medical Center, School of Medicine, Shanghai Jiao Tong University, Key Laboratory of Pediatric Hematology & Oncology of China Ministry of Health, Shanghai, China; 4 Department of Pediatrics, Qilu Hospital of Shandong University, Jinan, China; 5 Department of Hematology/Oncology, Children’s Hospital of Soochow University, Suzhou, China; 6 Department of Pediatrics, Key Laboratory of Birth Defects and Related Disease of Women and Children, Ministry of Education, West China Second University Hospital, Sichuan University, Chengdu, China; 7 Department of Hematology/Oncology, Children’s Hospital of Nanjing Medical University, Nanjing, China; 8 Department of Pediatrics, Union Hospital of Tongji Medical College, Huazhong University of Science and Technology, Wuhan, China; 9 Department of Hematology and Oncology, Second Affiliated Hospital of Anhui Medical University, Hefei, China; 10 Department of Pediatrics, Affiliated Hospital of Qingdao University, Qingdao, China; 11 Department of Hematology and Oncology, Children’s Hospital of Fudan University, Shanghai, China; 12 Department of Pediatrics, Xiangya Hospital Central South University, Changsha, China; 13 Department of Hematology, Kunming Children’s Hospital, Kunming, China; 14 Department of Pediatrics, Tongji Hospital of Tongji Medical College, Huazhong University of Science and Technology, Wuhan, China; 15 Department of Hematology/Oncology, Children’s Hospital Affiliated to Shanghai Jiao Tong University, Shanghai, China; 16 Department of Hematology/Oncology, Xi’an Northwest Women’s and Children’s Hospital, Xi’an, China; 17 Department of Pediatrics, Hong Kong Children’s Hospital, Chinese University of Hong Kong, Hong Kong, Hong Kong SAR, China

**Keywords:** acute lymphoblastic leukemia, CAR-T, children, immunotherapy, testicular leukemia

## Abstract

**Introduction:**

Testicular relapse constitutes one of the most frequent extramedullary recurrences in pediatric acute lymphoblastic leukemia (ALL), yet its clinical management remains incompletely characterized.

**Methods:**

This study assessed treatment outcomes and long-term survival in children with testicular relapse following initial therapy under the CCCG-ALL-2015 study (ChiCTR-IPR-14005706, http://www.chictr.org.cn). In total, 66 patients from 13 medical centers were retrospectively analyzed. Clinical characteristics and survival outcomes were compared across salvage treatment modalities.

**Results:**

The median interval from initial diagnosis to testicular relapse was 37 months. Among 59 patients who received post-relapse therapy, the 2-year overall survival (OS) rate was 86.1% after a median follow-up of 33 months. Patients treated with chimeric antigen receptor T-cell (CAR-T) therapy showed a 2-year OS of 90.7%, compared to 81.7% in those managed with conventional regimens, such as chemotherapy, orchiectomy, or hematopoietic stem-cell transplantation (P > 0.05). Among 37 children with isolated testicular relapse, 18 underwent CAR-T therapy and 10 underwent orchiectomy, achieving 2-year OS rates of 92.3% and 100%, respectively (P > 0.05).

**Discussion:**

Testicular relapse typically emerged approximately 3 years after initial diagnosis. CAR-T therapy proved to be both safe and effective, providing survival comparable to conventional regimens and offering potential advantages in preserving life quality among long-term survivors.

## Introduction

Acute lymphoblastic leukemia (ALL) is the most prevalent pediatric malignancy (1), accounting for a substantial proportion of childhood cancer diagnoses. Despite marked improvements in frontline therapy over recent decades, with 5-year overall survival (OS) rates exceeding 90% in select cohorts, the prognosis for patients who experience relapse remains poor (1, 2). The testes have long been regarded as a sanctuary site due to the blood-testis barrier, and consequently serve as the second most common site for extramedullary relapse in pediatric ALL. Although the application of contemporary intensive chemotherapy such as high-dose methotrexate has reduced the risk of testicular relapse to 2% or lower (3), it continues to pose a serious therapeutic challenge. Reported survival outcomes following isolated testicular relapse (ITR) vary widely, with OS estimates ranging from 53 to 84% (4, 5).

Management strategies following testicular relapse remain heterogeneous across studies. Salvage approaches have traditionally included systemic chemotherapy, orchiectomy, radiation, and hematopoietic stem-cell transplantation (HSCT). More recently, chimeric antigen receptor modified T-cell (CAR-T) therapy has emerged as a promising immunotherapeutic option, with preliminary reports suggesting efficacy in testicular involvement; however, data from large-scale cohorts and studies with extended follow-up remain scarce (6–11). Comparative analyses of these treatment modalities are limited, and the long-term benefits of orchiectomy in the era of advanced systemic therapy remain poorly defined. A clearer understanding of the therapeutic landscape is critical to optimizing outcomes in patients with testicular relapse.

In the present study, clinical features and treatment outcomes were retrospectively evaluated in children with testicular relapse of ALL from 13 medical centers across China. Survival rates were compared among patients receiving different salvage therapies to identify therapeutic strategies offering superior efficacy with reduced toxicity.

## Patients and methods

### Study design and patient cohort

This retrospective, multicenter study included 66 pediatric patients with testicular relapse of ALL treated across 13 medical centers in China. Inclusion criteria included the following: patients aged 0–18 years at initial diagnosis and enrolled in the Chinese Children’s Cancer Group study ALL‐2015 (CCCG‐ALL‐2015) clinical trial (12) between January 2015 and December 2019; B-lineage ALL (B-ALL) at initial diagnosis; testes as the initial site of relapse during the first complete remission (CR1), encompassing both ITR and testicular relapse combined with other sites (CTR). ITR was defined as histologically or ultrasonographically confirmed leukemic infiltration of enlarged testes, with a bone marrow (BM) blast count below 5% and no evidence of extramedullary involvement. CTR was defined as testicular enlargement accompanied by a BM blast count exceeding 5% and/or evidence of leukemic infiltration in other extramedullary sites. Clinical and biological characteristics, salvage therapy strategies, and outcomes were systematically collected and analyzed. Fusion gene status was determined by fluorescence *in situ* hybridization (FISH) and/or reverse transcription polymerase chain reaction (RT-PCR). Follow-up data were updated as of July 2023. This study was approved by the IRB of Shanghai Children’s Medical Center Affiliated Shanghai Jiao Tong University School of Medicine (Approval No. SCMCIRB-K2014060). Written informed consent was provided by the parents of all patients in compliance with the Declaration of Helsinki.

### Salvage therapy and outcome measures

Salvage treatment following testicular relapse varied among centers and was based on institutional protocols and patient-specific considerations. Patients were stratified into two groups according to salvage modality: CAR-T treated or traditional treated, including chemotherapy, orchiectomy, radiotherapy, HSCT, or combinations thereof. The primary endpoint was OS, defined as the time from first testicular relapse to death from any cause or last follow-up. The secondary endpoint was disease-free survival (DFS), defined as the time from first testicular relapse to the occurrence of second relapse, death from any cause, or last follow-up, whichever occurred first.

### Statistical analysis

Continuous variables were tested for normality with the Shapiro-Wilk test. Accordingly, they are expressed as mean ± standard deviation for normally distributed data or median with interquartile ranges (IQR) for non-normally distributed data. Categorical variables were reported as counts with corresponding proportions and compared using the Chi-square test or Fisher’s exact test. Survival distributions were estimated using the Kaplan-Meier method with 95% confidence intervals (CI), and differences between groups were assessed by the log-rank test. All statistical tests were two-sided, and *P* values below 0.05 were considered statistically significant. Analyses were conducted using SPSS v25.0 (IBM Corp, Armonk, NY, USA) and R v4.4.3 (http://www.r-project.org).

## Results

### Patient characteristics

**Figure 1:**
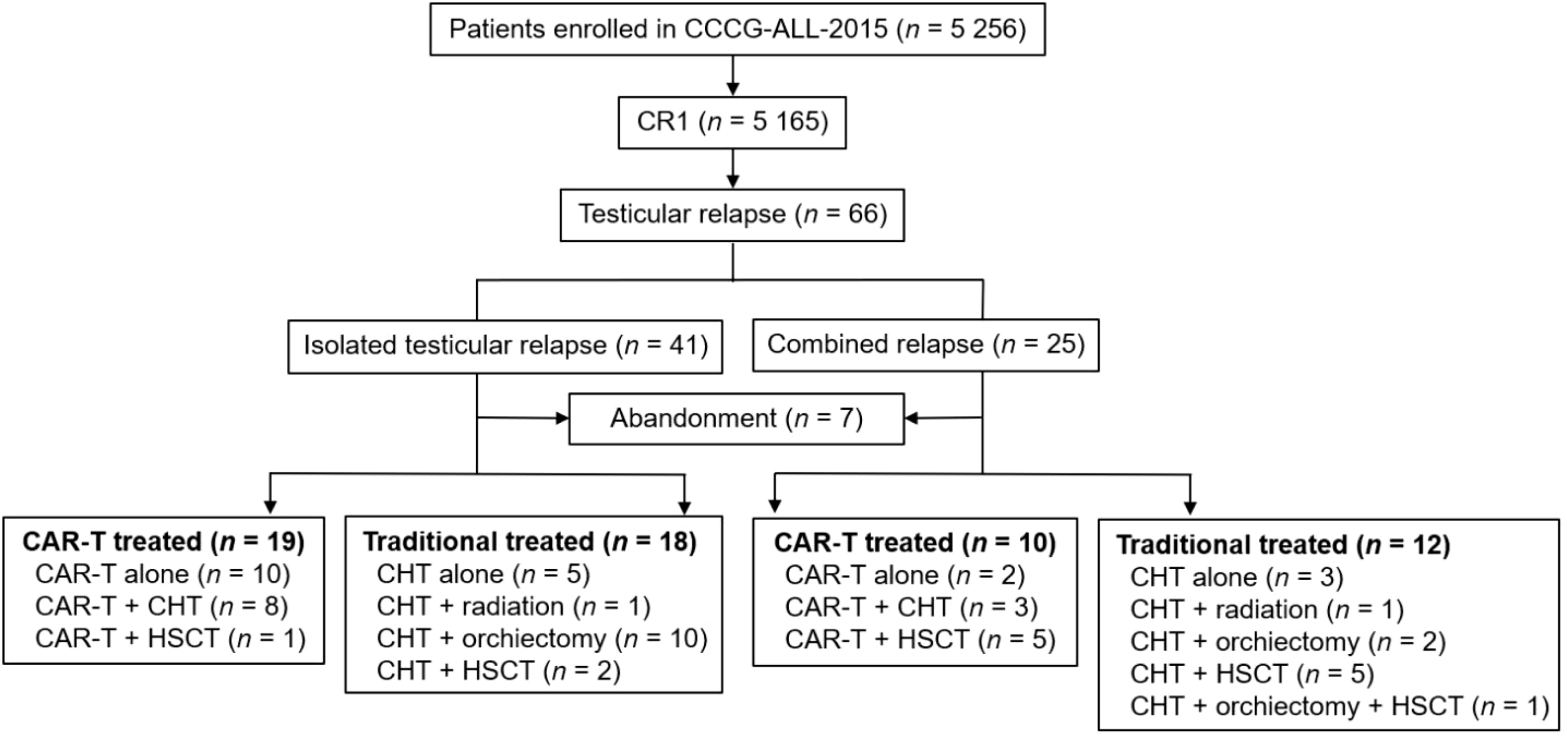
Study flow diagram. CR1, first complete remission; CAR-T, chimeric antigen receptor-modified T-cell therapy; CHT, chemotherapy; HSCT, allogeneic hematopoietic stem-cell transplantation.

The study flow diagram is presented in **Figure 1**. Among 5 256 newly diagnosed pediatric ALL cases enrolled in the CCCG-ALL-2015 study across 13 medical centers, testicular relapse was identified in 66 patients (1.3%). The number of patients recruited at each center is provided in **Supplementary Table S1**. Of these, 41 cases were diagnosed as ITR and 25 as CTR. Clinical and genetic characteristics of the 66 patients are detailed in **Table 1**. The median age at initial diagnosis was 3 years (IQR: 2–5). Fusion gene positivity was detected in 26 patients (39%), including 17 cases harboring ETV6::RUNX1. The median interval from initial diagnosis to testicular relapse was 37 months (IQR: 30.5–42).

**Table 1:** Clinical and genetic features of pediatric ALL patients with testicular relapse.

Characteristic	All patients *n* = 66	CAR-T treated *n = 29*	Traditional treated *n* = 30
Age at initial diagnosis, years	3 [2–5]	3 [2–4.5]	3 [2–5]
Initial risk group (CCCG-ALL-2015), *n* (%)			
Low risk	38 (58%)	19 (66%)	16 (53%)
Intermediate risk	28 (42%)	10 (34%)	14 (47%)
Fusion gene at initial diagnosis, *n* (%)			
Negative	40 (60%)	15 (52%)	21 (70%)
ETV6::RUNX1	17 (26%)	10 (34%)	6 (20%)
High-risk cytogenetics	9 (14%)	4 (14%)	3 (10%)
MLLr	5	2	3
Ph-like	2	2	0
E2A::PBX1	2	0	0
Age at testicular relapse, years	7 [5–8]	7.4 ± 2.7	5 [5–8]
Relapse time after diagnosis, months	37 [30.5–42]	40.7 ± 14.7	35.5 [31–40.3]
Relapse site, *n* (%)			
ITR	41 (62%)	19 (66%)	18 (60%)
CTR	25 (38%)	10 (34%)	12 (40%)
Testis +BM	22	9	10
Testis +CNS	2	1	1
Testis +BM+CNS	1	0	1
CAR-T cell type, *n* (%)			
CD19	18 (27%)	18 (62%)	–
CD19/22	11 (17%)	11 (38%)	–

Continuous variables were presented as mean ± SD or median (interquartile range, IQR) based on their distribution. ALL, acute lymphoblastic leukemia; BM, bone marrow; CNS, central nervous system; MLLr, MLL rearrangement; ITR, isolated testicular relapse; CTR, testicular relapse combined with other sites; CAR-T, chimeric antigen receptor-modified T-cell therapy.

### Treatment and outcomes following testicular relapse

Among the 66 patients with testicular relapse, seven discontinued treatment. The remaining 59 patients (89%) underwent salvage therapy and had evaluable follow-up data. The median follow-up was 33 months (IQR: 20-49). The 2-year OS and DFS rates were 86.1% (95% CI: 76.9%–96.3%) and 82.5% (95% CI: 72.7%–93.7%), respectively. Among the 59 patients who received salvage therapy after testicular relapse, one patient had missing MRD data at relapse. The remaining 58 patients were grouped according to MRD level at testicular relapse (< 0.01%, 0.01% to 5%, > 5%) and analyzed using the log-rank test for OS and DFS, as shown in **Supplementary Table S2**. MRD levels at testicular relapse were not significantly associated with outcome.

**Figure 2:**
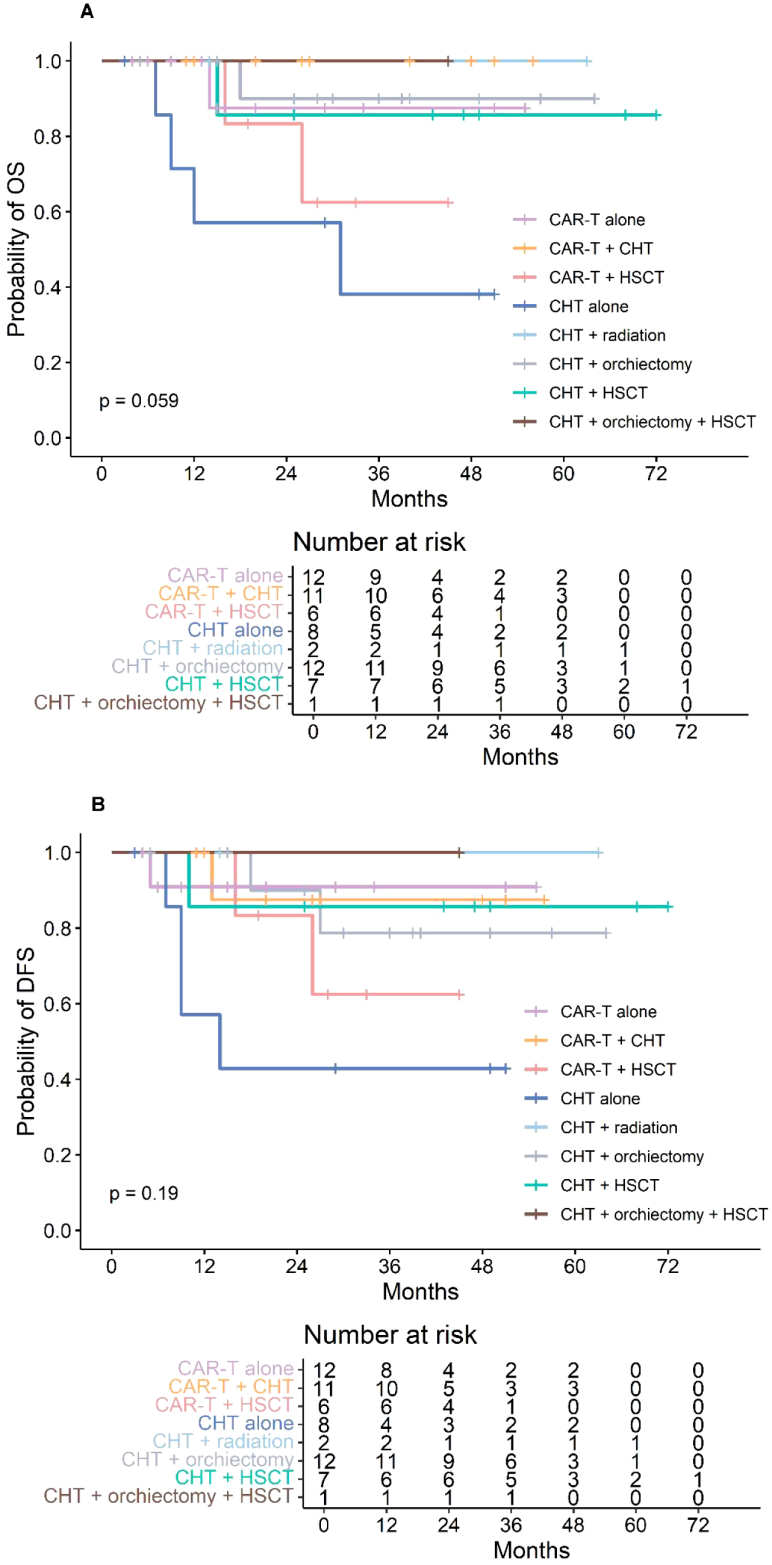
**(A)** Overall survival (OS) of 59 pediatric patients following testicular relapse. **(B)** Disease-free survival (DFS) of 59 pediatric patients following testicular relapse. CHT, chemotherapy; CAR-T, chimeric antigen receptor T-cell therapy; HSCT, allogeneic hematopoietic stem cell transplantation.

**Figure 3:**
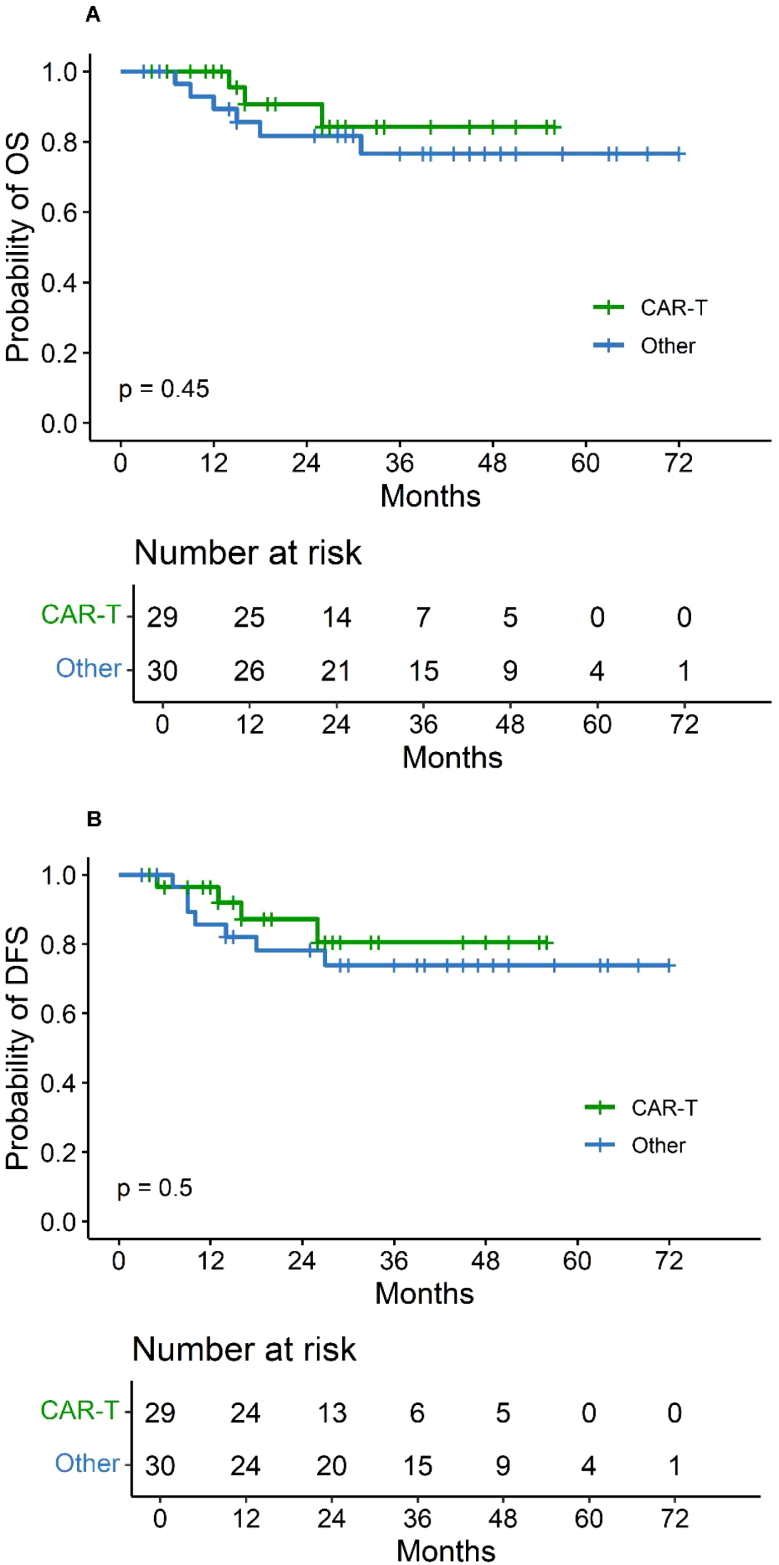
**(A)** OS stratified by salvage treatment modality in 59 pediatric ALL patients following testicular relapse. **(B)** DFS stratified by salvage treatment modality in the same cohort. CAR-T, chimeric antigen receptor T-cell therapy.

Post-relapse salvage therapy was heterogeneous (**Table 2**; **Figure 2**) and included CAR-T therapy in 29 patients (49%) and traditional regimens in 30 patients (51%). Median follow-up was 26 months (IQR: 15–40) in the CAR-T treated group and 43 months (IQR: 28–51) in the traditional treated group. Patients treated with CAR-T showed slightly higher survival rates, with 2-year OS of 90.7% (95% CI: 79.2%–100%) compared to 81.7% (95% CI: 68.3%–97.6%) in the conventional group (*P* = 0.45; **Figure 3A**), and 2-year DFS of 87.2% (95% CI: 74.5%–100%) versus 78.2% (95% CI: 64.2%–95.3%) (*P* = 0.5; **Figure 3B**), respectively. Within the CAR-T treated group, 18 patients received CD19-targeted CAR-T cells, and 11 patients received a coadministration of CD19- and CD22-targeted CAR-T cells. Patients treated with CD19/22 CAR-T showed slightly higher survival rates, with 2-year OS of 100% compared to 87.5% (95% CI: 72.7%–100%) in the CD19 CAR-T group (*P* = 0.29), and 2-year DFS of 100% versus 81.9% (95% CI: 65.2%–100%) (*P* = 0.18), respectively. For ITR patients treated with CD19 CAR-T (n = 12), the 2-year OS and DFS were 90.0% (95% CI: 73.2%–100%) and 91.7% (95% CI: 77.3%–100%), respectively. For CTR patients treated with CD19 CAR-T (n = 6), the 2-year OS and DFS were 83.3% (95% CI: 58.3%–100%) and 66.7% (95% CI: 37.9%–100%), respectively. In subgroup analysis by relapse site, no significant differences in the 2-year OS or DFS were observed between the CD19 CAR-T group and CD19/22 CAR-T group (all *P* > 0.05). Cytokine release syndrome (CRS) occurred in 9 (31.0%) patients, was grade 1 in eight patients, and was grade 3 in one patient with ITR. Grade 1 CRS was more common in those with ITR compared with patients with CTR (7 of 19 vs 1 of 10 patients, *p* = 0.201 in *Fisher’s* test). No patient experienced grade 4 CRS. Grade 3 seizure developed in 2 (6.9%) patients, both of whom had ITR. No local effect was observed. Within the traditional treated cohort, patients were further stratified into five subgroups: “chemotherapy alone”, “chemotherapy + irradiation”, “chemotherapy + orchiectomy”, “chemotherapy + HSCT”, and “chemotherapy + orchiectomy + HSCT”, as detailed in **Table 2**; **Figure 2**. No significant differences in the 2-year OS or DFS were observed between the CAR-T group and any of the traditional regimen groups (all *P* > 0.05). Subsequent events following relapse included second relapse, death, and loss to follow-up. Among the nine patients who died, eight (89%) deaths were due to disease progression, while one (11%) death was attributed to infection.

**Table 2:** Salvage therapy following testicular relapse in pediatric ALL patients.

Salvage therapy	All, *n* = 59			ITR, *n* = 37			CTR, *n* = 22		
	*n* (%)	2-year OS KM% (95% CI)	2-year DFS KM% (95% CI)	*n* (%)	2-year OS KM% (95% CI)	2-year DFS KM% (95% CI)	*n* (%)	2-year OS KM% (95% CI)	2-year DFS KM% (95% CI)
CAR–T	29 (49)	90.7 (79.2–100)	87.2 (74.5–100)	19 (51)	92.9 (80.3–100)	94.7 (85.2–100)	10 (45)	87.5 (67.3–100)	75.0 (50.3–100)
CAR-T alone	12	87.5 (67.3–100)	90.9 (75.4–100)	10	85.7 (63.3–100)	90.0 (73.2–100)	2	100	100
CAR-T + CHT	11	100	87.5 (67.3–100)	8	100	100	3	100	50.0 (12.5–100)
CAR–T + HSCT	6	83.3 (58.3–100)	83.3 (58.3–100)	1	100	100	5	80.0 (51.6–100)	80.0 (51.6–100)
Traditional regimen	30 (51)	81.7 (68.3–97.6)	78.2 (64.2–95.3)	18 (49)	94.1 (83.6–100)	88.2 (74.2–100)	12 (55)	63.6 (40.7–99.5)	63.6 (40.7–99.5)
CHT alone	8	57.1 (30.1–100)	42.9 (18.2–100)	5	75.0 (42.6–100)	50.0 (18.8–100)	3	33.3 (6.7–100)	33.3 (6.7–100)
CHT+ radiation	2	100	100	1^a^	–	–	1	100	100
CHT + orchiectomy	12	90.0 (73.2–100)	90.0 (73.2–100)	10	100	100	2^b^	–	–
CHT + HSCT	7	85.7 (63.3–100)	85.7 (63.3–100)	2	100	100	5	80.0 (51.6–100)	80.0 (51.6–100)
CHT + orchiectomy + HSCT	1	100	100	0			1	100	100

OS, overall survival; DFS, disease-free survival; CHT, chemotherapy; CAR-T, chimeric antigen receptor-modified T-cell therapy; HSCT, allogeneic hematopoietic stem-cell transplantation; ITR, isolated testicular relapse; CTR, testicular relapse combined with other sites.

a The patient with ITR who received chemotherapy and radiation remained alive and event-free, and was followed up for 14 months.

b Of the two patients with CTR who received chemotherapy and orchiectomy, one died from disease progression at 18 months, while the other remained alive and event-free and was followed up for 5 months.

#### CAR-T versus orchiectomy as salvage therapy for ITR

Among 37 patients with ITR, 18 (49%) received CAR-T therapy without subsequent HSCT. Of these, 10 received CAR-T alone and eight received CAR-T in combination with chemotherapy. After a median follow-up of 20 months (IQR: 13–48), one patient experienced relapse involving both BM and kidney at 5 months post-relapse and died within 14 months. The resulting 2-year OS and DFS rates were 92.3% (95% CI: 78.9%–100%) and 94.4% (95% CI: 84.4%–100%), respectively. One additional patient who received HSCT following CAR-T therapy remained event-free through the end of follow-up. Ten patients (27%) underwent orchiectomy combined with chemotherapy as salvage treatment for ITR. The median follow-up in this group was 36 months (IQR: 28–49). One patient experienced BM relapse at 27 months and was subsequently lost to follow-up, while the remaining nine patients survived event-free to the end of follow-up, with a 2-year OS and DFS of 100%. No significant differences in survival outcomes were observed between patients receiving CAR-T without HSCT and those undergoing orchiectomy-based salvage therapy.

#### CAR-T for CTR

Among the 22 patients with CTR, 10 (45%) received CAR-T therapy, with or without subsequent HSCT. The median follow-up was 28 months (IQR: 19–40). All five patients treated with CAR-T alone remained alive, including one patient who developed secondary ITR at 13 months and was successfully salvaged with HSCT. Of the five patients who underwent CAR-T followed by HSCT, two died from disease progression.

## Discussion

In this study, testicular relapse occurred in 1.3% of pediatric ALL patients initially treated under the CCCG-ALL-2015 protocol across 13 medical centers in China, aligning with previous reports that estimate its incidence at 2% or lower (3). The median interval between initial diagnosis and relapse was 37 months, consistent with prior studies indicating that testicular relapse typically arises more than 6 months after completion of frontline therapy (13). Although the occurrence of testicular relapse has become increasingly uncommon with contemporary treatment protocols, its clinical management remains variable and insufficiently characterized across studies (3). Conventional salvage therapies following testicular relapse, including chemotherapy, testicular irradiation, orchiectomy, and HSCT, have historically constituted the mainstay of treatment, while CAR-T therapy has recently emerged as a promising immunotherapeutic approach. Selection of therapeutic modality often depends on whether the testicular relapse is isolated or concurrent with other sites, yet comparative evaluations of efficacy among these treatment strategies remain limited and incompletely defined.

For patients with ITR, salvage regimens combining chemotherapy with orchiectomy or local irradiation have been commonly applied in previous studies (3, 14, 15). The REC80-ITR protocol, which incorporates both systemic chemotherapy and testicular irradiation, achieved 4-year OS and DFS of 67.7% and 41%, respectively (15). Although no conclusive evidence indicates that orchiectomy provides superior outcomes to testicular irradiation, it remains an appropriate option for patients with unilateral testicular involvement or those declining local irradiation (3, 14). HSCT has also been employed for isolated extramedullary relapse, yielding reported 10-year OS and DFS rates of 67% and 65%, respectively (16). However, fertility impairment remains a major concern, as procedures such as orchiectomy and testicular irradiation, as well as exposure to high cumulative doses of alkylating agents exceeding 4 000 mg/m^2^ cyclophosphamide equivalents or total body irradiation prior to HSCT, can adversely affect reproductive function (3, 17, 18). These limitations underscore the urgent need for novel therapeutic strategies that achieve durable remission while preserving fertility in children with testicular relapse.

CAR-T therapy has recently been explored as an innovative strategy for managing testicular relapse (6–8). Yu et al. first applied CAR-T therapy in ITR, demonstrating potent cytotoxic activity against leukemic infiltration in the testes without apparent impairment of normal testicular function (8). Subsequent clinical investigations have reported encouraging outcomes. In our previous study, CAR-T therapy was applied to treat seven B-ALL patients with ITR, yielding a 1-year event−free survival (EFS) of 83.3% (6). Wang et al. reported a 1-year EFS of 95% in a cohort of 20 ITR patients receiving CAR-T therapy (7). These findings suggest that CAR-T therapy represents a promising and potentially less toxic alternative for patients with ITR. In the present study, 18 patients with ITR received CAR-T therapy without subsequent HSCT, achieving 2-year OS and DFS rates of 92.3% and 94.4%, respectively. One patient who underwent HSCT following CAR-T therapy remained event−free at the end of follow-up. Ten patients treated with orchiectomy exhibited 2−year OS and DFS rates of 100%. Survival outcomes did not differ significantly between the orchiectomy and CAR-T cohorts (both *P > 0.05*).

Treatment strategies for patients with concurrent testicular and BM relapse are similar to those employed for isolated BM relapse (3). In this cohort, approximately half of the patients with CTR underwent allogeneic HSCT, showing a trend toward improved outcomes (**Supplementary Figure S1**). However, no statistically significant difference in 2-year OS or DFS was observed, potentially due to limited follow-up duration and the favorable prognosis among patients treated with CAR-T therapy. Notably, all five patients with CTR who received CAR-T without subsequent HSCT remained alive, including one who later developed secondary ITR and achieved remission following HSCT. These findings suggest that HSCT may serve as a viable salvage option for patients experiencing post-CAR-T recurrence, although further clinical evidence is needed to substantiate its role in this context.

This study has several limitations, including its retrospective design, modest sample size, and heterogenous cohort, making it difficult to draw definite conclusions. Assessment of sexual function and gonadal hormone levels was not feasible because of the young age of participants, highlighting the need for continued long-term follow-up to evaluate potential reproductive outcomes. Despite these constraints, this investigation represents the largest multicenter retrospective analysis to date examining clinical characteristics and therapeutic outcomes of ALL patients with testicular relapse in China, encompassing the largest patient cohort and longest observation period following CAR-T-based salvage therapy. Epperly et al. and Holland et al. underscored the necessity of systematic evaluations using advanced imaging techniques, such as ^18^Fluorodeoxyglucose (^18^F-FDG) positron emission tomography/computed tomography (PET/CT), to evaluate the efficacy of CAR-T in extramedullary disease (10, 11). Future studies should incorporate PET/CT to evaluate the efficacy of CAR-T for testicular relapse. At the same time, future work should also pay attention to patients’ mental states, quality of life, and the prognostic impact of B-cell aplasia.

## Conclusion

This study shows that testicular relapse occurred in 1.3% of pediatric ALL patients and mainly occurred 3 years after initial diagnosis, which calls for more attention to the relapse in testes during long-term follow-up. For testicular relapse, CAR-T was effective, which might have an advantage in improving life-quality of survivors. CAR-T without subsequent HSCT holds the promise for achieving durable survival in patients with ITR. Prospective studies are needed to confirm these findings.

## Data Availability

The original contributions presented in the study are included in the article/**Supplementary Material**. Further inquiries can be directed to the corresponding authors.
