# Supplementary material for: Efficacy of chimeric antigen receptor T-cell therapy in testicular relapse of pediatric acute lymphoblastic leukemia: a multicenter retrospective study
Source: Front Immunol. 2026 Feb 11;17:1766494. doi: 10.3389/fimmu.2026.1766494 (PMC12932491; doi:10.3389/fimmu.2026.1766494)

**Table S1**. Number of patients at each center

| Medical center | Number of patients recruited |
|---|---|
| Institute of Hematology & Blood Diseases Hospital, Chinese Academy of Medical Sciences & Peking Union Medical College, Tianjin, China | 16 |
| Shanghai Children's Medical Center, Shanghai Jiao Tong University School of Medicine, Shanghai, China | 14 |
| Qilu Hospital of Shandong University, Jinan, China | 9 |
| Children's Hospital of Soochow University, Suzhou, China | 6 |
| West China Second University Hospital, Chengdu, China | 5 |
| Children's Hospital of Nanjing Medical University, Nanjing, China | 3 |
| Union Hospital of Tongji Medical College, Huazhong University of Science and Technology, Wuhan, China | 3 |
| Second Affiliated Hospital of Anhui Medical University, Hefei, China | 3 |
| Affiliated Hospital of Qingdao University, Qingdao, China | 2 |
| Children's Hospital of Fudan University, Shanghai, China | 2 |
| Xiangya Hospital Central South University, Changsha, China | 1 |
| Kunming Children's Hospital, Kunming, China | 1 |
| Tongji Hospital of Tongji Medical College, Huazhong University of Science and Technology, Wuhan, China | 1 |

**Table S2**. Association between MRD level at testicular relapse and patients' survival outcomes

| | All, *n* = 58 | | | ITR, *n* = 36 | | | CTR, *n* = 22 | | |
|---|---|---|---|---|---|---|---|---|---|
| | ***n*** | **2-year OS KM% (95% CI)** | **2-year DFS KM% (95% CI)** | ***n*** | **2-year OS KM% (95% CI)** | **2-year DFS KM% (95% CI)** | ***n*** | **2-year OS KM% (95% CI)** | **2-year DFS KM% (95% CI)** |
| MRD | | | | | | | | | |
| < 0.01% | 31 | 88.6 (77.2–100) | 89.0 (77.9–100) | 29 | 92.3 (82.5–100) | 92.7 (83.5–100) | 2[b] | 50.0 (12.5–100) | 50.0 (12.5–100) |
| 0.01% to 5% | 13 | 90.0 (73.2–100) | 80.0 (58.7–100) | 7 | 100 | 80.0 (51.6–100) | 6 | 80.0 (51.6–100) | 80.0 (51.6–100) |
| > 5% | 14 | 76.2 (55.8–100) | 68.4 (46.9–99.7) | 0 | | | 14 | 76.2 (55.8–100) | 68.4 (46.9–99.7) |
| *P* value [a] | | 0.5 | 0.43 | | 0.63 | 0.83 | | 0.81 | 0.94 |

*Abbreviations: OS: overall survival; DFS: disease-free survival; ITR: isolated testicular relapse; CTR: testicular relapse combined with other sites; MRD: minimal residual disease measured by flow cytometry; BM: bone marrow.*

[a] *Differences in survival among MRD strata (< 0.01%, 0.01% to 5%, > 5%) were assessed using the log-rank test.*

[b] *These two patients had testicular relapse combined with central nervous system relapse.*

**Figure S1**. Overall survival (OS, A) and disease-free survival (DFS, B) of 22 pediatric ALL patients after testicular relapse combined with other sites, by HSCT as salvage therapy.

Abbreviations: HSCT, allogeneic hematopoietic stem cell transplantation.

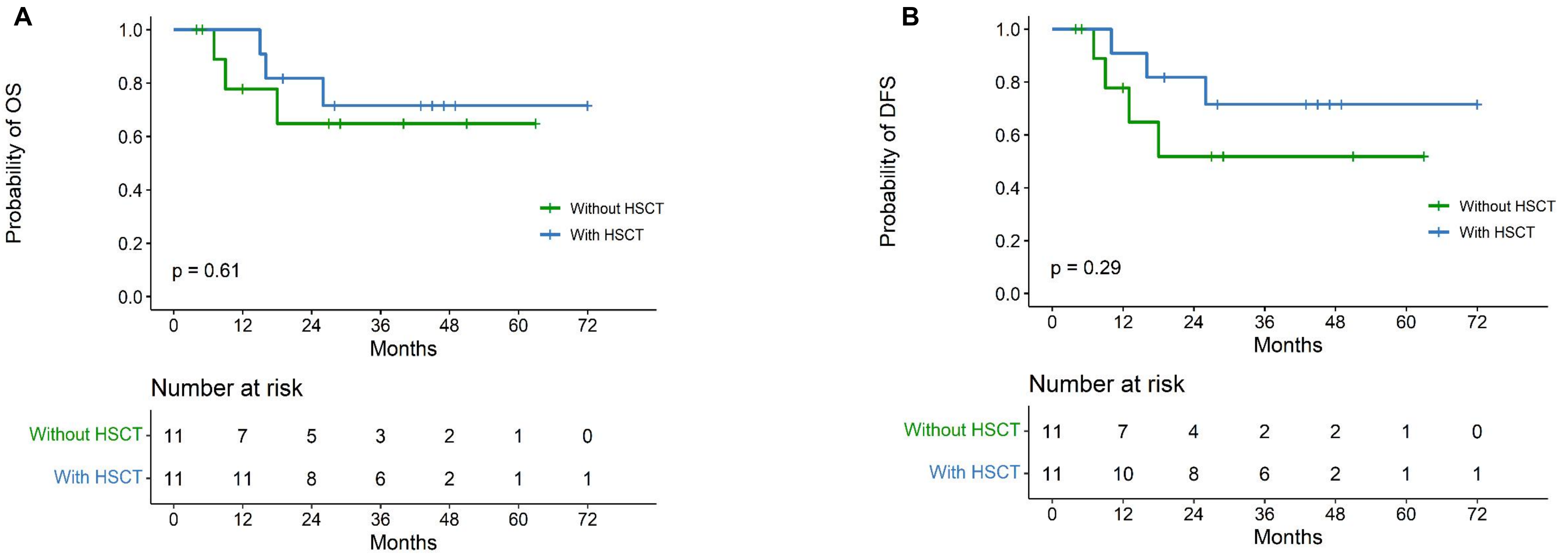

Supplement: Supplementary file 1 [file DataSheet1.pdf]
